# Nicotinic Activity of Arecoline, the Psychoactive Element of "Betel Nuts", Suggests a Basis for Habitual Use and Anti-Inflammatory Activity

**DOI:** 10.1371/journal.pone.0140907

**Published:** 2015-10-21

**Authors:** Roger L. Papke, Nicole A. Horenstein, Clare Stokes

**Affiliations:** 1 Department of Pharmacology and Therapeutics, University of Florida, PO Box 100267 Gainesville, Florida, 32610–0267, United States of America; 2 Department of Chemistry, University of Florida, PO Box 117200, Gainesville, Florida, 32611–7200, United States of America; Duke University Medical Center, UNITED STATES

## Abstract

Habitual chewing of "betel nut" preparations constitutes the fourth most common human self-administration of a psychoactive substance after alcohol, caffeine, and nicotine. The primary active ingredient in these preparations is arecoline, which comes from the areca nut, the key component of all such preparations. Arecoline is known to be a relatively non-selective muscarinic partial agonist, accounting for many of the overt peripheral and central nervous system effects, but not likely to account for the addictive properties of the drug. We report that arecoline has activity on select nicotinic acetylcholine receptor (nAChR) subtypes, including the two classes of nAChR most related to the addictive properties of nicotine: receptors containing α4 and β2 subunits and those which also contain α6 and β3 subunits. Arecoline is a partial agonist with about 6–10% efficacy for the α4* and α6* receptors expressed in *Xenopus* oocytes. Additionally, arecoline is a silent agonist of α7 nAChR; while it does not activate α7 receptors when applied alone, it produces substantial activation when co-applied with the positive allosteric modulator PNU-120696. Some α7 silent agonists are effective inhibitors of inflammation, which might account for anti-inflammatory effects of arecoline. Arecoline's activity on nAChR associated with addiction may account for the habitual use of areca nut preparations in spite of the well-documented risk to personal health associated with oral diseases and cancer. The common link between betel and tobacco suggests that partial agonist therapies with cytisine or the related compound varenicline may also be used to aid betel cessation attempts.

## Introduction

**Fig 1:**
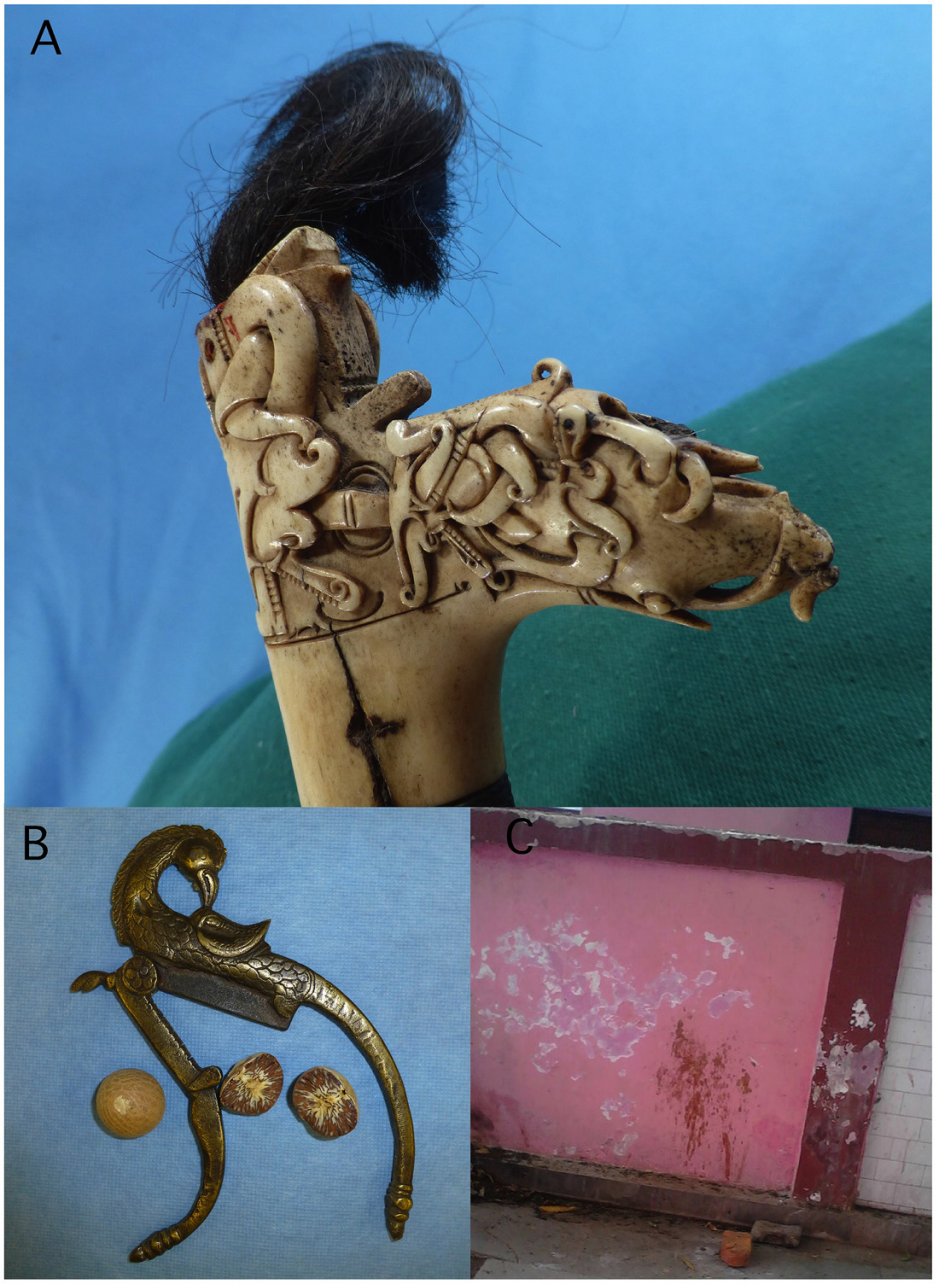
The world of betel nuts. (A) The carved hilt of a Dayak head hunter sword. The chewing of areca nut is an important an essential element of the culture of these tribes on the Island of Borneo, and has been throughout their known history. It was reflection on this abstract, almost psychedelic, carving that inspired the senior author (RLP) to investigate the activity of areca nuts and arecoline. (B) An areca nut and traditional nut cutter, an essential piece of the paraphernalia associated with this drug habit. (C) A wall in Barabanki, Uttar Pradesh, India stained by the expectorate of a betel chewer. Photo by permission Ashok Kumar.

"Bloody Mary's chewing betel nuts…" For many Westerners, these lines from Rogers and Hammerstein's musical is the extent of their knowledge of the fourth most common drug habit in the world, after alcohol, nicotine and caffeine [1]. The psychoactive agents associated with this habit come from the seed of the *Areca catechu* palm, which is sliced, often combined with spices, and wrapped in leaves of the vine *Piper betle* that have been spread with slaked lime, making packets suitable for chewing known as "betel quids". Much of the history and culture of South Asia involves the use of areca nuts, from hallucinogenic carvings of Dyak headhunter swords (Fig 1A), to the elegant accoutrements for use of betel quids (Fig 1B). It is still common to see sidewalks and walls besmirched with crimson betel spittle in poor neighborhoods of India and parts of Asia (Fig 1C). The details of preferred areca preparations vary significantly across Asia, and in recent decades have included some form of tobacco for about 50% of the users [1], although the key ingredient is always areca nut.

There are numerous alkaloids in areca nuts, the predominant psychoactive agent being arecoline [2], a muscarinic acetylcholine receptor agonist [3–5]. While some effects of the areca nut are obvious, most notably the copious production of bright red saliva, others are more subtle and, as with nicotine, rely on subjective accounts. Euphoric or anxiolytic effects are reported, as well as both sedation and arousal, and there are frequent historical references to betel as an aphrodisiac [6,7]. These effects most likely account for the short-term reinforcing aspects of the drug use, and dependence and mild withdrawal have frequently been reported. However, what might underlie the addictive properties of betel use has been unclear. Historically, habitual areca use was socially acceptable, even expected, in Asian cultures, but now betel use is transitioning to the situation where it is tolerated but no longer widely encouraged. In large measure this change in public attitudes has come from appreciation of the health liability associated betel use, especially in regard to oral diseases and cancers [8–10].

While tobacco use is rightly maligned for all of the associated health risks, nicotine receptors are being assigned new potential roles as therapeutic targets, especially in regard to cognitive disorders [11–13] and, more recently, inflammatory diseases and pain [14,15]. Likewise, there may be therapeutic directions suggested by the properties of arecoline that are not due to muscarinic agonist activity. There have been numerous reports of anti-inflammatory or immunosuppressant activity associated with arecoline [4,16,17]. Interestingly, it has also been suggested that betel nut use may be of therapeutic value for schizophrenia [18,19]. These are both areas in which α7 nAChR have recently been identified as a new therapeutic target, and α7-targeting drugs that have low efficacy for ion channel activation, such as the weak partial agonist GTS-21, appear to be among the most promising candidates [12,20–23]. Indeed, in regard to inflammation and pain, agents recently identified as α7 "silent agonists" [24,25], which produce no significant channel activation but may regulate intracellular signal transduction, show better therapeutic potential than typical agonists such as nicotine [21,26,27]. Although silent agonists produce little or no channel activation, they induce desensitized conformations similar to those produced by efficacious agonists, which can be confirmed by co-application with a positive allosteric modulator (PAM) such as PNU-120596, which converts those desensitized states into very actively conducting states. We therefore investigated the effects of areca and arecoline on α7 nAChR and extended those studies to identify a likely mechanism for betel addiction.

## Materials and Methods

### Commercial reagents

Acetylcholine chloride (ACh), arecoline, muscarine, methacholine, oxotremorine, carbachol, mecamylamine, and atropine were purchased from Sigma-Aldrich Chemical Company (St. Louis, MO). Fresh ACh stock solutions were made in Ringer's solution each day of experimentation. Stock solutions of the test drugs were made in Ringer's solution and kept at 4°C and used within two days. Working solutions were prepared freshly at the desired concentration from the stored stock.

### Areca nut infusion

Whole *Areca catechu* nuts were purchased through eBay from Rider International Health Foods, Nuts & More, Chicago IL. The dry areca nuts, weighing approximately 10 g each, were broken into small bits (roughly 0.5 cm cubes) with a hammer and pruning shears, then pulverized in an electric coffee grinder. The rough powder was added to Ringer's solution (pH 7.2) at 200 mg per ml. The mixture was stirred 10 minutes at room temperature, then drip-filtered with P8 coarse filter paper. The pH was measured at this point to be 5.4 and was then brought up to 7.2.

### Heterologous expression of nAChRs in *Xenopus laevis* oocytes

Human nAChR clones and concatamers were obtained from Dr. J. Lindstrom (University of Pennsylvania, Philadelphia, PA). The human resistance-to-cholinesterase 3 (RIC-3) clone, obtained from Dr. M. Treinin (Hebrew University, Jerusalem, Israel), was co-injected with α7 to improve the level and speed of α7 receptor expression without affecting the pharmacological properties of the receptors [28]. Subsequent to linearization and purification of the plasmid cDNAs, cRNAs were prepared using the mMessage mMachine in vitro RNA transfection kit (Ambion, Austin, TX).

Oocytes were surgically removed from mature *Xenopus laevis* frogs (Nasco, Ft. Atkinson, WI) and injected with appropriate nAChR subunit cRNAs as described previously [29]. Frogs were maintained in the Animal Care Service facility of the University of Florida, and all procedures were approved by the University of Florida Institutional Animal Care and Use Committee. In brief, the frog was first anesthetized for 15–20 min in 1.5 L frog tank water containing 1 g of 3-aminobenzoate methanesulfonate buffered with sodium bicarbonate. The harvested oocytes were treated with 1.25 mg/ml collagenase (Worthington Biochemicals, Freehold, NJ) for 2 h at room temperature in calcium-free Barth’s solution (88 mM NaCl, 1 mM KCl, 2.38 mM NaHCO_3_, 0.82 mM MgSO_4_, 15 mM HEPES, and 12 mg/l tetracycline, pH 7.6) to remove the follicular layer. Stage V oocytes were subsequently isolated and injected with 50 nl of 5–20 ng nAChR subunit cRNA. Recordings were carried out 1–7 days after injection.

### Two-electrode voltage clamp electrophysiology

Experiments were conducted using OpusXpress 6000A (Molecular Devices, Union City, CA) [29]. Both the voltage and current electrodes were filled with 3 M KCl. Oocytes were voltage-clamped at -60 mV. The oocytes were bath-perfused with Ringer’s solution (115 mM NaCl, 2.5 mM KCl, 1.8 mM CaCl_2_, 10 mM HEPES, and 1 μM atropine, pH 7.2) at 2 ml/min for α7 receptors and at 4 ml/min for other subtypes. To evaluate the effects of experimental compounds compared to ACh-evoked responses of various nAChR subtypes expressed in oocytes, baseline conditions were defined by two initial applications of ACh made before test applications. The solutions were applied from a 96-well plate via disposable tips, and the test compounds were applied alone, co-applied with ACh, or co-applied with PNU-120596. For the concentration-response study, drug applications alternated between ACh controls and experimental compounds. Unless otherwise indicated, drug applications were 12 s in duration followed by a 181 s washout period for α7 receptors and 6 s with a 241 s washout for other subtypes. A typical recording for each oocyte constituted two initial control applications of ACh, an experimental compound application, and then a follow-up control application of ACh to determine the desensitization or rundown of the receptors. The control ACh concentrations were 60 μM for α7, 100 μM for α3β4, and 30 μM for α4β2. The responses of α4β2 and α3β4-expressing cells were measured as peak current amplitudes, and the α7 data were calculated as net charge, as previously described [30].

Data were collected at 50 Hz, filtered at 20 Hz, analyzed by Clampfit 9.2 (Molecular Devices) and Excel 2003 (Microsoft, Redmond, WA), and normalized to the averaged peak current or net-charge response of the two initial ACh controls for each oocyte [30]. Data were expressed as means ± SEM from at least four oocytes for each experiment and plotted by Kaleidagraph 3.0.2 (Abelbeck Software, Reading, PA). Multi-cell averages were calculated for comparisons of complex responses. To permit better comparisons between experiments each single cell response was normalized to the average of the two initial controls obtained from that cell. Averages of the normalized data were calculated for each of the 10,500 points in each of the 210 s traces (acquired at 50 Hz), as well as the standard errors for those averages.

## Results

**Fig 2:**
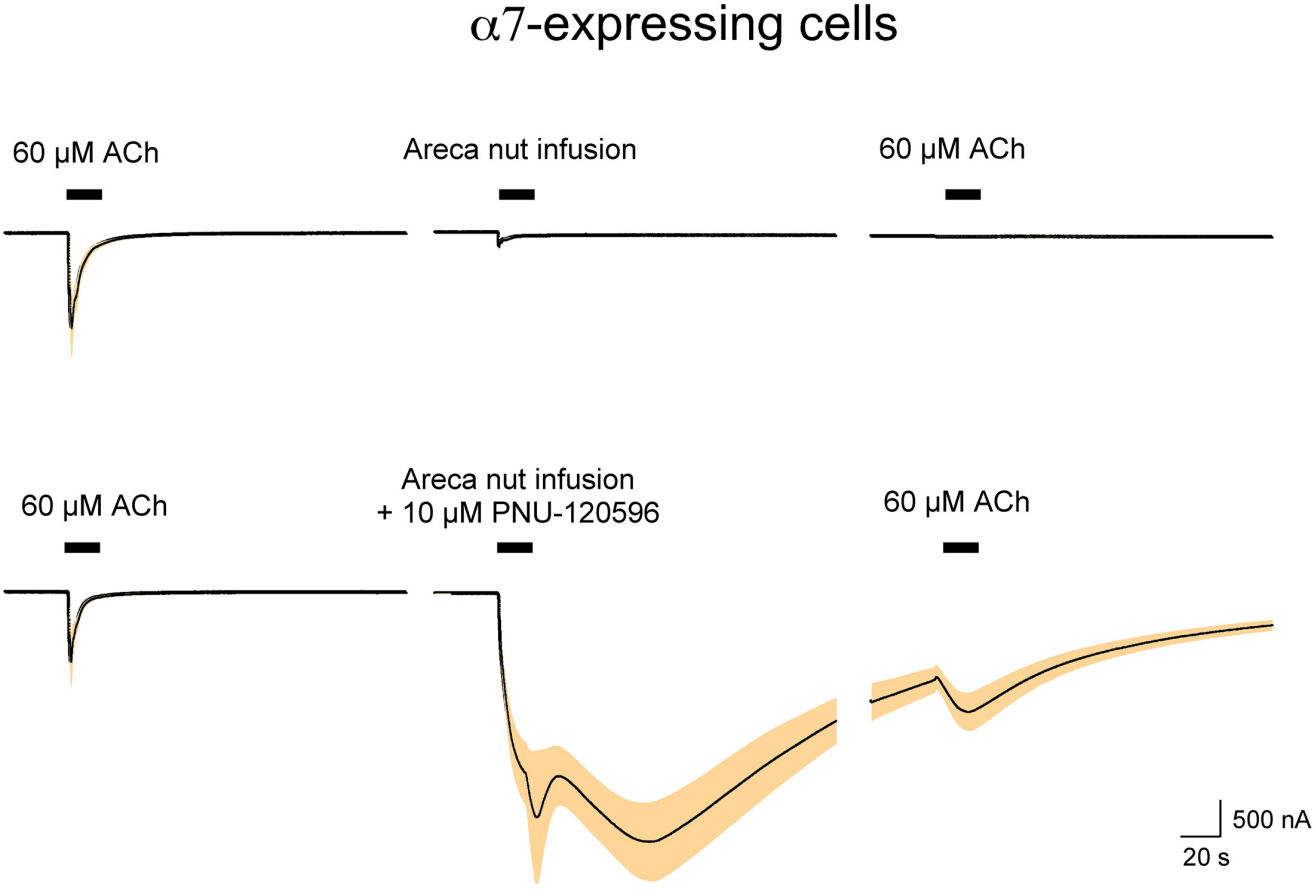
The effects of areca nut infusion (see Methods) on oocytes expressing α7 nAChR. Cells were initially tested for their responses to control applications of 60 μM ACh prior to the application of the filtered nut infused solution. After a 4-minute wash, the infusion solution ± 10 μM PNU-120596 was applied (0.4 ml over 12 seconds) followed by another application of 60 μM ACh. The cells were voltage clamped at -60 mV, and the traces shown represent the average response (black line) ± the S.E.M. (shaded band) calculated for each of the 10,500 points in the 210 s traces (acquired at 50 Hz). For application of the infusion solution alone n = 8, and for the data obtained in the presence of PNU-120596 (n = 5 cells).

An aqueous extract of areca nut (areca nut infusion, ANI) was prepared as described above, and after obtaining control ACh responses ANI was applied to oocytes expressing human α7 nAChR. ANI alone evoked minimal responses compared to ACh but suppressed subsequent responses to ACh (Fig 2). ANI produced no detectable responses in oocytes that were not injected with RNA for nAChR subunits (not shown). When 10 μM of the α7-selective PAM PNU-120596 [31] was added to the ANI, the α7-expressing cells showed responses that were much larger than those evoked by ACh alone. PNU-120596 is known to destabilize desensitized states of α7 nAChR and so typically evokes responses that are much more prolonged than those stimulated by ACh or other α7 agonists [32]. When used in combination with ACh in our system, PNU-120596-potentiated responses normally decay well back to baseline during the normal washout procedure [32]. However, potentiated ANI responses were biphasic and only partially decayed through the washout period. When 60 μM ACh was applied 4 minutes after the initial ANI application, there was a transient increase in current added to the still decaying responses to the previous application of ANI plus 10 μM PNU-120596.

**Fig 3:**
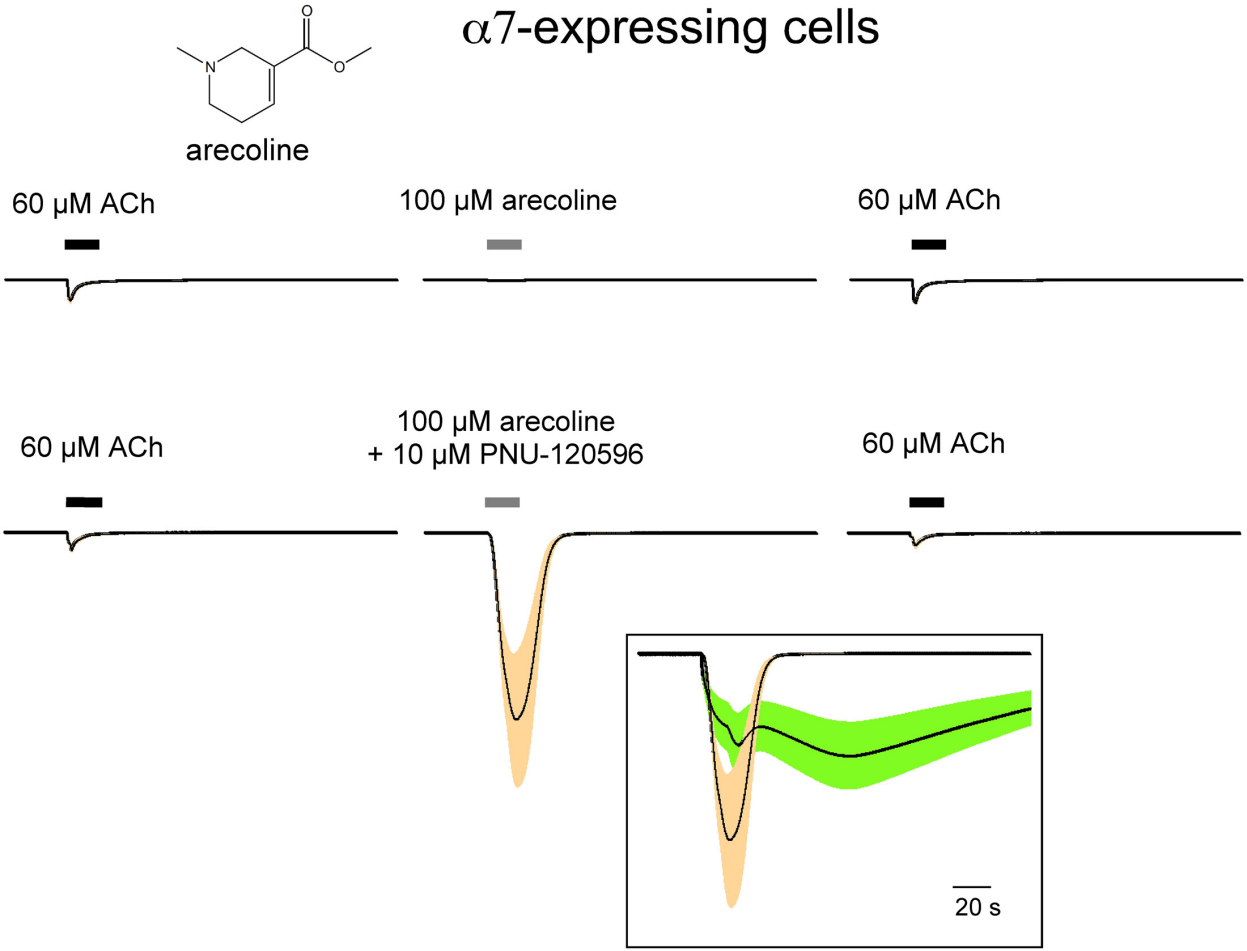
Effects 100 μM arecoline (structure illustrated) on oocytes expressing α7 nAChR. Cells were tested for their responses to control applications of 60 μM ACh prior to the application of the test solution. The second of two such control applications is shown. After a 4 minute wash period, 100 μM arecoline ± 10 μM PNU-120596 was applied (0.4 ml over 12 seconds) followed by another application of 60 μM ACh, as shown. Prior to the calculation of the multi-cell averages, each single cell response was normalized to the average of the two initial controls obtained from that cell. The cells were voltage clamped at -60 mV, and the traces shown represent the average of the normalized responses (black line) ± the S.E.M. (shaded band) calculated for each of the 10,500 points in the 210 s traces (acquired at 50 Hz). For arecoline alone (n = 8), and for arecoline plus PNU-120596 (n = 7). In order to allow for comparison between experiments, the data for the responses to the nut infusion plus PNU-120596 shown in Fig 2 were also normalized to their respective controls and are displayed along with the arecoline plus PNU-120596 data in the insert.

There are many potentially active molecules in the areca nut, including numerous alkaloids, with the most abundant and active for producing responses in central and peripheral nervous system tissues being arecoline, a muscarinic agonist with activity at M1, M2, and M3-type receptors. Arecoline is a tertiary amine (Fig 3) with good brain penetration, as evidenced by its numerous central nervous system effects [7]. Therefore we tested arecoline specifically for its effects on α7 nAChR. As shown in Fig 3, 100 μM arecoline applied alone did not produce significant activation of α7 nAChR. Subsequent ACh-evoked responses were also largely unaffected. However, when arecoline was co-applied with 10 μM PNU-120596, responses had a peak amplitude 9.6 ± 3.4 times larger than the ACh controls (n = 7), with net charge 37 ± 10 times larger. In order to facilitate comparisons, the responses evoked by ANI plus PNU-120596 shown in Fig 2 were normalized to their ACh controls and are shown in the insert of Fig 3 compared to the PNU-120596-potentiated 100 μM arecoline responses. The arecoline responses had a larger peak amplitude, but, as typical for the potentiated responses of ACh, they decayed more rapidly and completely back to the original baseline. The ACh-evoked responses following the 100 μM arecoline applications (± PNU-120596) were not significantly different from the original ACh-evoked controls. These data indicate that arecoline is an α7 silent agonist [24,25], essentially ineffective at activating the ion channel through the normal orthosteric agonist binding site, but able to induce the non-conducting conformational states that are destabilized by PNU-120596.

**Fig 4:**
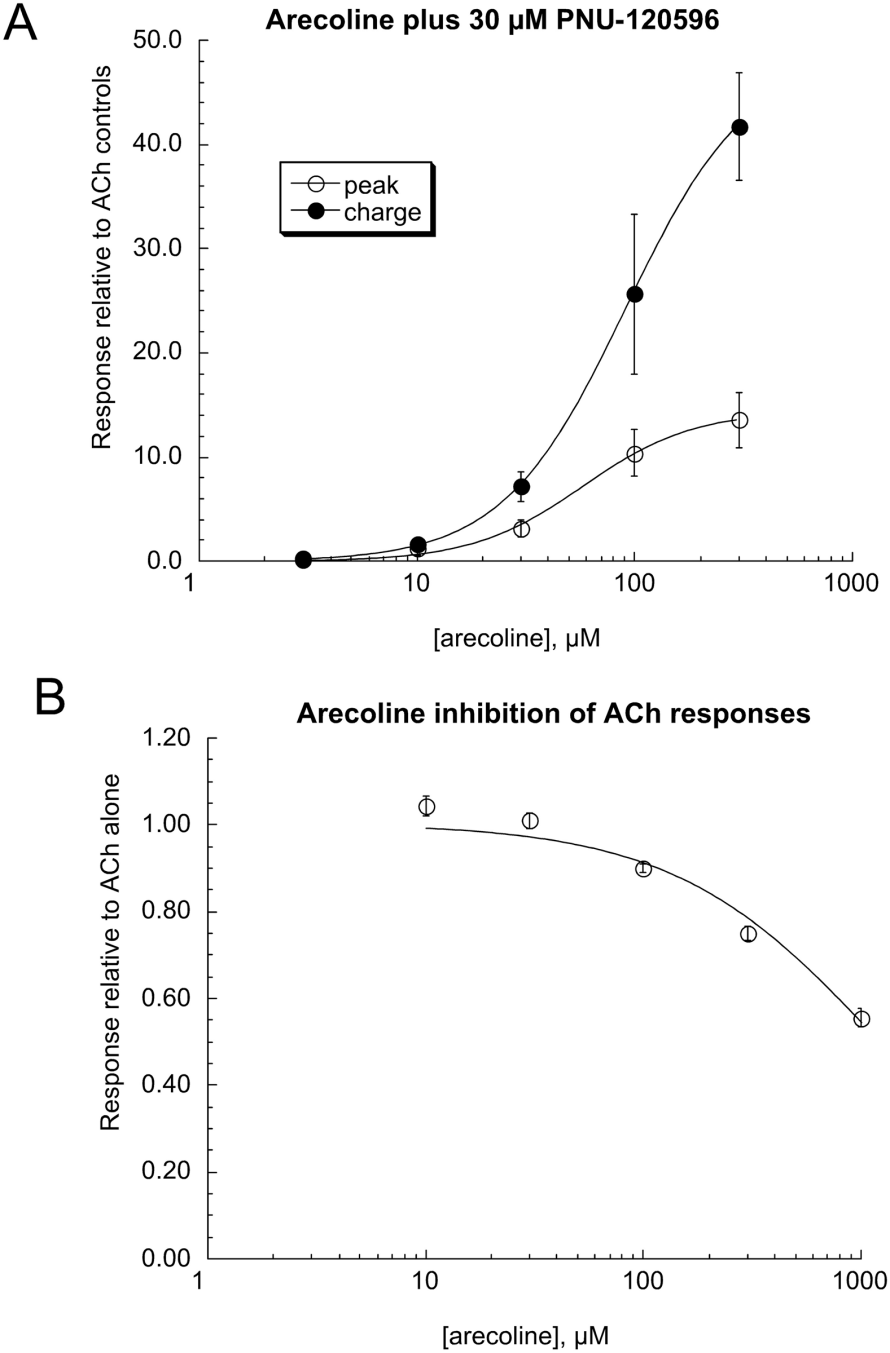
Arecoline concentration-response studies. (A) Oocytes expressing α7 were tested with co-applications of 30 μM PNU-120596 plus varying concentrations of arecoline. Both peak currents and net charge responses were calculated and normalized to the average of two initial 60 μM ACh control responses in the same cells. The EC_50_ values were 60 ± 7 and 93 ± 2 μM for peak currents and net charge, respectively. Relative to ACh controls, the I_max_ values were 15 ± 1 and 49 ± 1 for peak currents and net charge, respectively. (B) Since in the absence of a PAM, silent agonists can function as antagonists of typical agonists, the potency of arecoline for antagonizing 60 μM ACh-evoked responses was tested. Arecoline was surprisingly ineffective at inhibiting ACh responses, with an IC_50_ > 1000 μM.

A concentration-response study of arecoline responses potentiated by 30 μM PNU-120596 (Fig 4A) indicated EC_50_ values of 60 ± 7 and 93 ± 2 μM for peak currents and net charge, respectively. As we have previously reported [24,25,27], in the absent of a PAM, silent agonists can function as antagonists of ACh-evoked responses. However, as shown in Fig 4B, with a simple co-application protocol arecoline had very low potency for inhibiting 60 μM ACh-evoked responses (IC_50_ > 1000 μM). This is consistent with the hypothesis that some structural requirement features of silent agonists may be distinct from those for effective binding and inhibition at the ACh (i.e. orthosteric) binding site [25].

**Fig 5:**
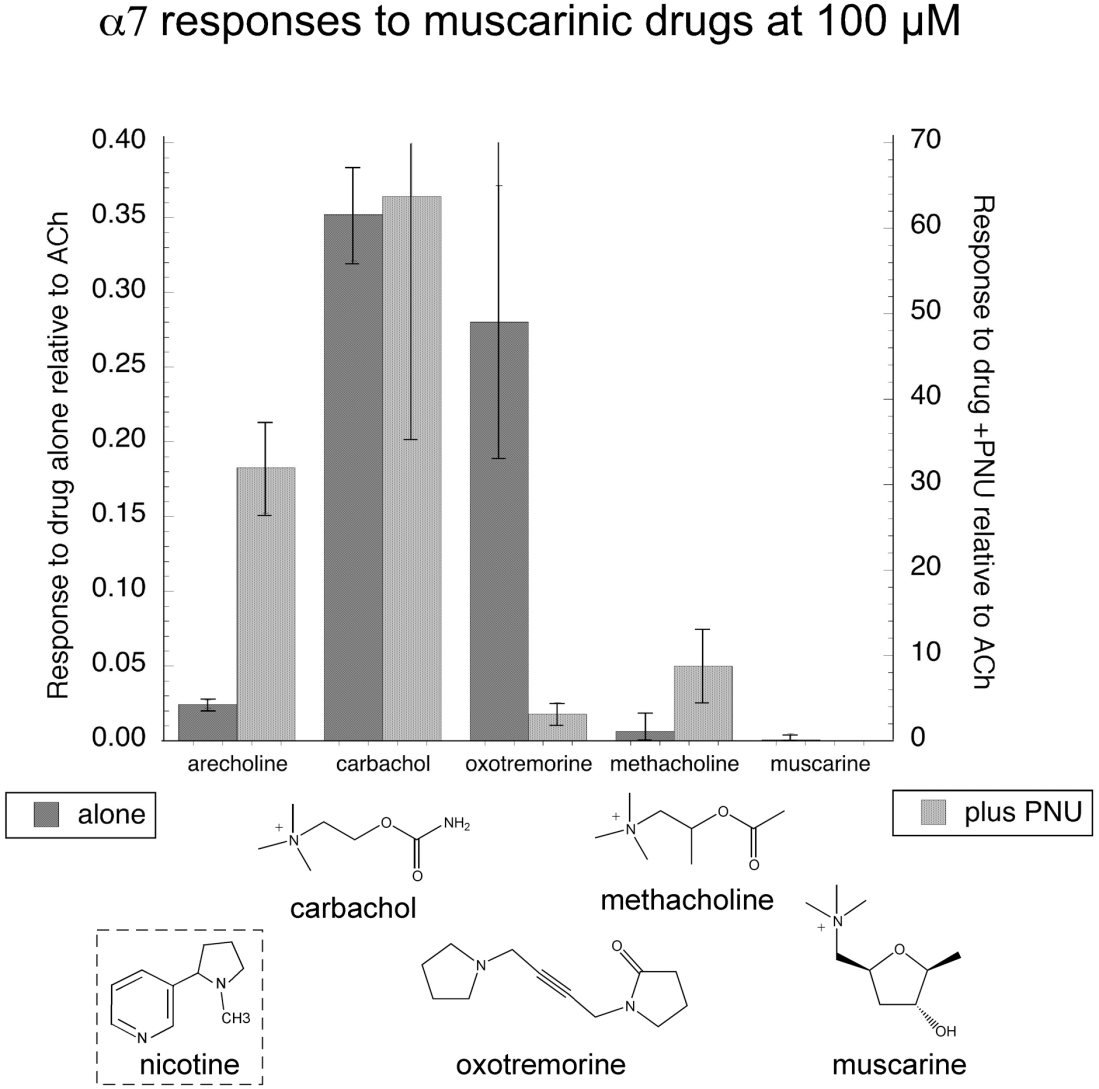
Agonist and silent agonist activity of muscarinic cholinergic agonists. The pharmacophore for silent agonism of α7 is distinct from that for activation in the absence of a PAM [25]. Since arecoline is known to be a muscarinic agonist, we tested additional compounds with muscarinic activity for their ability to activate α7 in the absence and presence of 10 μM PNU-120596. The structures of the test compounds are shown, as well as, that of nicotine for comparison.

In order to further investigate the hypothesis that there may be common elements shared by the pharmacophores of muscarinic agonists and α7 silent agonists, we tested other known activators of muscarinic AChR, including the non-selective agonist carbachol, with and without PNU-120596. As shown in Fig 5, carbachol stimulated α7 receptors under both conditions. Interestingly, oxotremorine, while able to produce small but significant activation in the absence of the PAM, produced relatively little additional activation in the presence of PNU-120596. Although not as active as arecoline, methacholine was also a silent agonist for α7, while muscarine failed to activate the receptors under either condition.

This small panel of muscarinic compounds is structurally diverse. Arecoline most resembles carbachol and methacholine in terms of the relative distance between the positive charge and the hydrogen bond acceptor. It differs from these two in terms of not having a “hard” quaternary ammonium charged center, but this appears not to be a factor given that methacholine may be classified as a silent agonist, while oxotremorine can not. Oxotremorine is in fact unusual for being able to produce significant orthosteric activation with relatively little potentiation by PNU-120596. This is in contrast to the usual case, as with carbachol, where effective orthosteric activation predicts effective allosterically potentiated activation. The orthosteric agonism of carbachol is not exhibited by the relatively homologous compound methacholine, and may be a reflection of conformational biasing of the methyl group when bound to the nAChR in the absence of the PAM.

**Fig 6:**
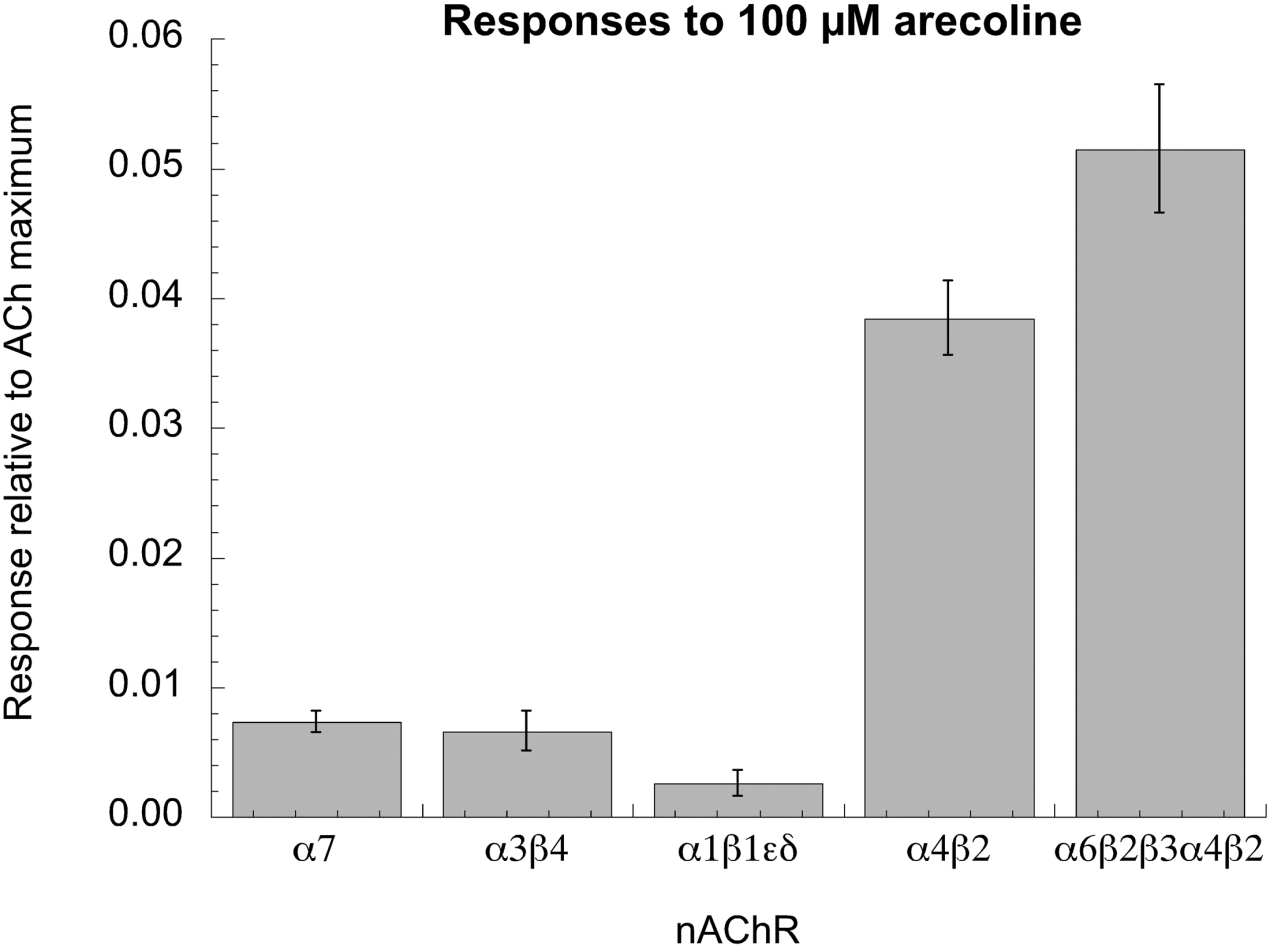
Arecoline activation of other nAChR subtypes. 100 μM arecoline was applied to cells expressing the nAChR subunits indicated. Responses of human α3β4, α7, and mouse muscle (α1β1εδ) subunits were barely at the threshold of detection, less than 1% the ACh maximum, extrapolated from comparisons to ACh controls and ACh concentration-response studies conducted previously. The responses of cells expressing α4β2 or a concatamer containing α6 and β3 in addition to α4 and β2 were substantially larger and well above the threshold of detection.

We also tested whether arecoline was able to activate other nAChR subtypes. As shown in Fig 6, although arecoline showed very little activity at the ganglion and muscle-type analogs (α3β4 and α1β1εδ, respectively), it showed small but significant activity with oocytes injected with α4β2 and the α6-containing concatamer (α6β2β3α4β2) [33].

Note that in the experiment shown in Fig 6, cells were injected with monomeric forms of α4 and β2 which is known to produce a heterogeneous population of receptors with two different ratios of α4 and β2 subunits. By co-expressing a β2-α4 concatamer with monomeric α4 or β2 subunits, it is possible to obtain homogeneous populations of receptors with defined subunit composition, either α4(3)β2(2) or α4(2)β2(3), respectively [34]. Based on the potency of ACh and nicotine for activating these receptors, they have been characterized as low sensitivity (LS) or high sensitivity (HS) subtypes. Chronic nicotine exposure has been shown to specifically upregulate the HS form of α4β2. This effect is believed to be important for the development of nicotine addiction and dependence. The expression of α6 and β3 subunits is high, and largely restricted to, dopaminergic neurons believed to mediate the chemical reward promoting nicotine self-administration (i.e. smoking). Therefore α4- and α6-containing receptors are considered important targets for smoking cessation therapies.

**Fig 7:**
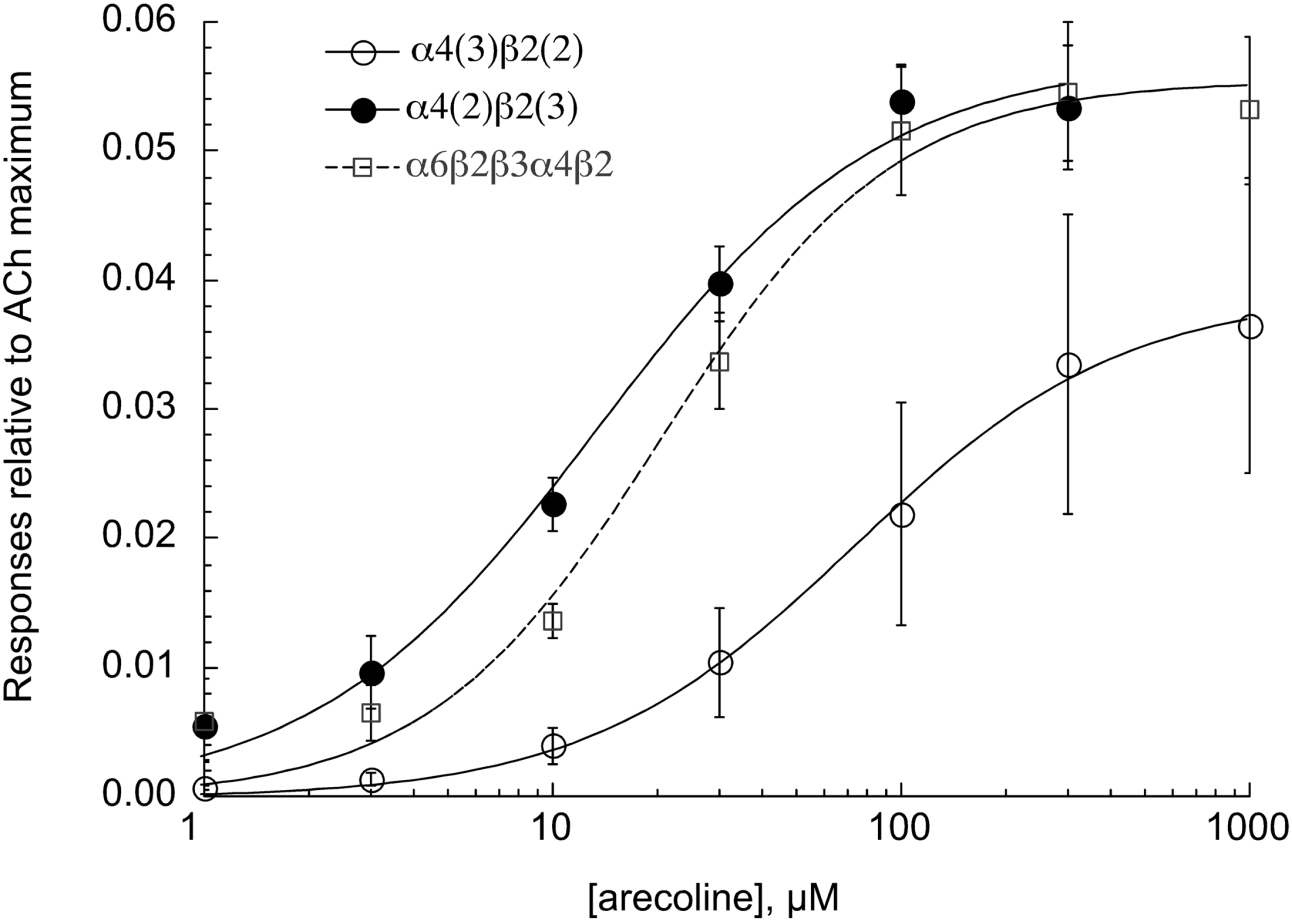
Arecoline concentration-response studies of α4- and α6-containing receptors. Data are the averages of at least 5 cells for each subtype: the α6-containing receptor produced with an α6β2β3α4β2 concatamer [33] and the high sensitivity (HS, α4(2) β2(3)) and low sensitivity (LS, α4(3) β2(2)) α4β2 nAChR produced with the β2-α4 concatamer and monomers. Responses were calculated relative to ACh control responses measured four minutes prior to the arecoline applications and then adjusted for the ratio between the ACh controls and ACh maximum responses determined in previous experiments. EC_50_s were 14 ± 3, 21 ± 4, and 75 ± 7 μM for HS α4β2, α6-containing, and LS α4β2 receptors, respectively.

**Fig 8:**
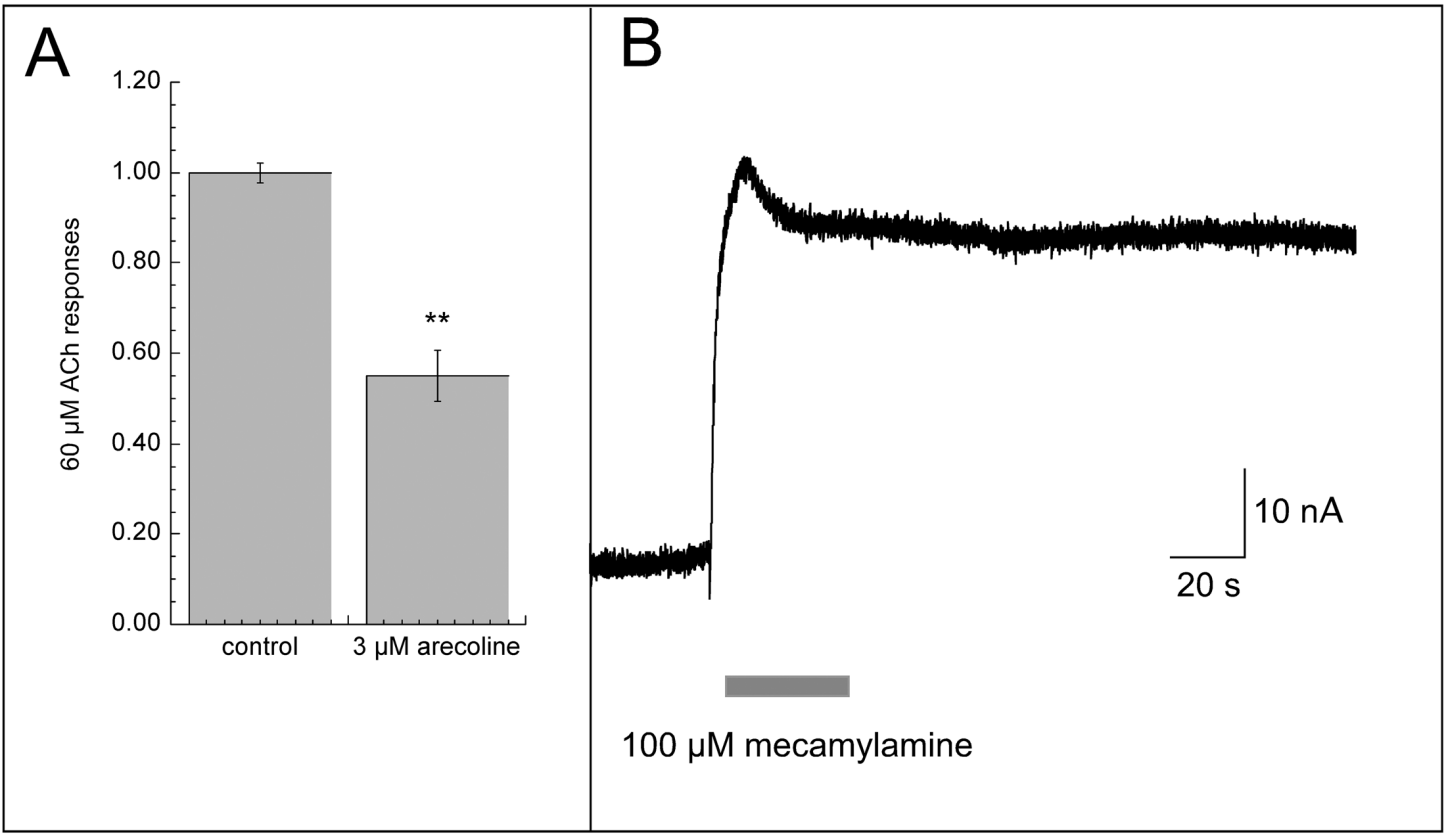
Modulation of HS α4β2 receptors with a low concentration of arecoline. (A) Partial agonists for α4β2 nAChR such as varenicline and cytisine [35] modulate the sensitivity of the receptors to the endogenous activator ACh through pre-desensitization for prolonged periods, even when present at very low concentrations. They can also stimulate low levels of tonic activation [35]. After obtaining initial control responses to ACh, a steady flow of 3 μM arecoline was applied to the bath. After 8 minutes the responses to a control application of ACh was reduced approximately 50%. (B) The perfusion of 3 μM arecoline was continued, and 100 μM mecamylamine was applied along with the arecoline to reveal the mecamylamine-sensitive steady-state current. The decrease in inward current shown is the averaged response of seven cells. Normalized relative to initial ACh controls and adjusted for ACh maximum, these currents indicated steady-state activation of approximately 1% ACh maximum.

As shown in Fig 7, arecoline is a relatively potent, albeit low efficacy, partial agonist for HS α4β2 and α6-containing receptors, suggesting that activity at these receptors may subtly mediate some of the reward associated with habitual betel use, and moreover may account for some of the addictive properties of areca. The typical pattern of betel use involves prolonged chewing the prepared quid and so would be expected to produce prolonged delivery of arecoline and other factors at low levels. This sort of presentation of a partial agonist will have two effects: it will down-regulate the phasic activity of other stimuli, such as oscillations in endogenous ACh, and it may also promote low levels of steady-state activation as receptors pass in and out of desensitized states [35,36]. As shown in Fig 8, the prolonged bath application of 3 μM arecoline to cells expressing HS α4β2 receptors produced substantial inhibition of ACh response and a steady-state current that was sensitive to the nAChR antagonist mecamylamine. This smoldering current was small but non-trivial, as it was approximately equal to 1% of the maximal transient current associated with ACh activation. The time-integrated effect of this activation could be substantial.

**Fig 9:**
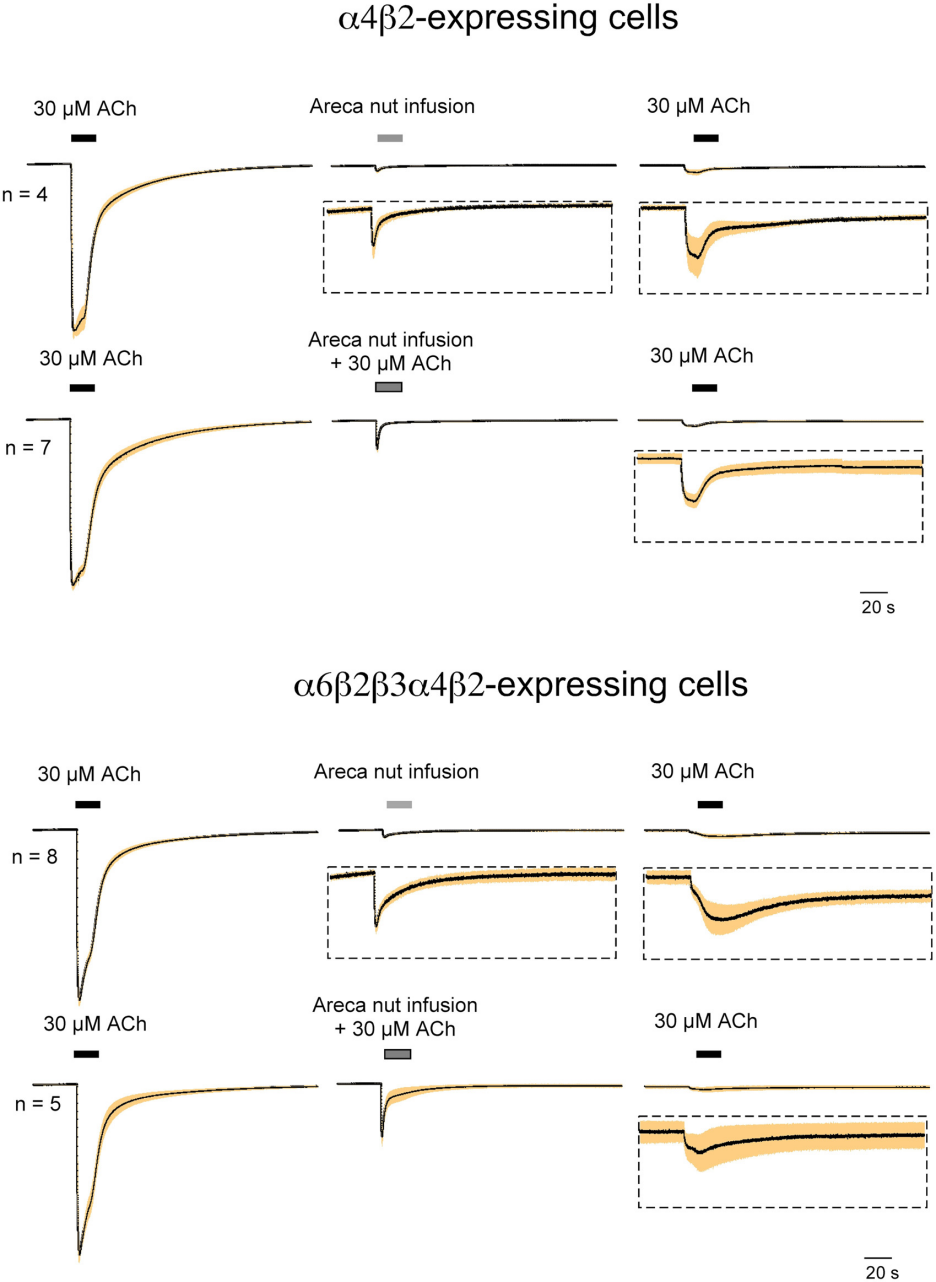
Heteromeric AChR sensitivity to areca nut infusion. Areca nut infusion was prepared as described above. The traces were scaled to ACh controls in each cell prior to calculating averages and S.E.M. The α4β2 receptors were formed from the co-expression of monomers. The infusion was applied either alone or was co-applied with ACh. The inserts below some of the traces display the same data scaled up by a factor of 10, and the traces shown represent the average of the normalized responses (black line) ± the S.E.M. (shaded band) calculated for each of the 10,500 points in the 210 s traces (acquired at 50 Hz). Consistent with the arecoline data, the infusion produced small activation of the nAChR and inhibited the responses to ACh at the control concentration. Consistent with effects of the nut infusion of α7 receptors in the absence of PNU-120596 (Fig 3), there was a profound inhibition of subsequent ACh-evoked responses.

The effects of areca nut infusion on α7 receptors were only partially mimicked by arecoline, so we also tested ANI on cells expressing α4β2 nAChR (mixed populations formed from monomers) or the α6-containing concatamer. As expected, the application of ANI produced small transient activation of both these receptor subtypes (Fig 9), and, similar to the effects on α7 receptors, there was a large inhibition of subsequent ACh-evoked responses. ANI co-application also antagonized the transient activation of these receptors by ACh.

## Discussion

Human behavior is linked to central nervous system reward circuits that can be manipulated by drugs, in turn promoting addictive behaviors that can be detrimental to our health or social well being. It has been known since the 1980s that there were specific receptors in the brain that were likely to mediate the addicting effects of tobacco [37], and connecting these receptors to the stimulation of mesolimbic dopamine neurons provided the association between smoking behavior and addiction [38]. However, even for nicotine, it has remained an open question whether the true underpinning of nicotine dependence is receptor activation or desensitization [39].

Our results show that areca nut use is accessing the nicotinic receptor systems of the body in several ways that may account its effects on human behavior, as well as previously reported effects on the immune system. There was strong α7 silent agonist activity in the raw areca nut infusion that could largely be explained by the presence of arecoline, and this activity was observed within a physiologically relevant range of arecoline concentration, although the effects of the extract were more protracted. The α7 silent agonist NS6740 also has prolonged effects on the conformational states of α7, inducing very stable desensitization lasting many minutes after a single application [26], although activations produced by co-applications of NS6740 with PNU-120596 are relatively transient when measured with the same methodology used for these experiments. It is therefore unclear why the potentiated effects of the infusion were so prolonged. Betel chewers typically achieve salivary concentrations of arecoline ranging 40 to 400 μM, and 90% of betel chewers in a recent study showed residual levels of at least 400 nM prior to chewing [40], even though their reported use was in most cases less than daily. We have not endeavored to measure the actual arecoline concentration in our infusion, so it is possible that it was above a plateau for maximal activation, and activity was sustainable as concentrations fell. It is also possible that there were additional effects from other alkaloids or compounds present in areca [2].

The discovery of arecoline's α7 silent agonist activity not only provides possible insight in to the mechanism for previously described effects on immune cells [4,16,17,41–46], but also provides an opportunity to refine models for the pharmacophore of α7 silent agonism in regard to comparisons with the other muscarinic activators tested.

While some α7 silent agonists may have significant therapeutic potential in their own right [26], arecoline would not be a good candidate for further development in that direction, due to its strong muscarinic activity and potential involvement with the carcinogenic effects of areca use. What is of much more significance for understanding areca as a drug of abuse is the selective partial agonist activity for α4- and α6- containing nAChR.

Several different classes of key nicotinic AChR subtypes arise from the expression of specific genes that code for these pentameric receptors, which are usually highlighted as mediators of ligand-gated ion channel function [47]. One important class contains the homomeric α7 receptors, which are found throughout the brain and body and often in nonneuronal cells such as those of the immune system. A second crucial class has the five different genes that have been identified to contribute subunits to the nicotinic receptors of the neuromuscular junction (α1, β1, γ, δ and ε). These muscle-type nAChR are the essential mediators of every human action, and to paraphrase Lord Sherrington, from whispering a syllable to felling a forest.

The entire autonomic nervous system relies on a third class of ganglionic nAChR that principally contains α3 and β4 subunits. A fourth class, associated with various heteromeric nAChR subtypes in the brain, serves diverse functions and mainly modulates activity of other neurotransmitter systems [48]. Receptors containing both α4 and β2 subunits are found throughout the brain, and their levels, as well as specific subunit composition, are regulated by nicotine exposure. It has been shown that β2-containing receptors are required for the acquisition of nicotine addiction [49]. As mentioned previously, partnered with the α4β2 receptors in dopaminergic neurons are also receptors containing α6 and β3 subunits, making these two types of receptors key targets for managing nicotine addiction and dependence [50].

There are two stages to the process of drug taking behavior leading to addiction. The first stage involves short-term "reinforcing" effects which promote the drug taking in a naive user. The second stage involves the development of dependence which leads to craving and ultimately withdrawal. It seems unlikely that arecoline/areca has reinforcing effects mediated by the low level of nAChR activation produced. It is more likely that short-term reinforcement of betel use is associated with the muscarinic "high" or intoxication. However, our data suggest that habitual use of areca will also work on the same receptors as does nicotine. This may lead to dependence and promote craving and withdrawal if is areca use is discontinued. Indeed, areca users attempting to quit manifest withdrawal symptoms similar to those of smokers, including irritability, mood swings, paranoia, anxiety, lack of concentration, and sleep disturbance [51].

The best smoking cessation therapies currently available are the β2 receptor partial agonists cytisine [52,53] and varenicline [54]. The efficacy of these compounds for the β2-containing receptors is similar to that of arecoline (Table 1), and like arecoline, these compounds can blunt the phasic activation of α4 and α6- containing receptors by ACh and other more efficacious agonists such as nicotine. At low concentration they can also produce low levels of steady-state activation [35], which may affect dopaminergic tone and diminish craving. These observations suggest that these smoking cessation agents could also be applied as a replacement therapy for betel users willing to quit their habit, motivated by social or health-related concerns.

**Table 1:** Partial agonist I_max_ values relative to Ach.

Receptor	cytisine	varenicline	arecoline
α4(3)β2(2)	0.10 ± 0.02^a^	0.08^b^	0.036 ± 0.003
α4(2)β2(3)	≤ 0.05 ^a^	0.13^b^	0.054 ± 0.004
α6β2β3α4β2	0.11 ± 0.03^c^	0.18 ± 0.11^c^	0.056 ± 0.003

^a^ [55]

^b^ [56]

^c^ [33]

This possible path for new addiction therapies opens the question of whether other commonalities exist between areca and tobacco use, for example, whether arecoline, like nicotine, can function as a chemical chaperone and upregulate nAChR expression and function in the brain. However, our data also make it clear that there is more to areca than arecoline, for example in regard to the functional down-regulation produced by the infusion that was not evident with arecoline alone.

While largely unknown in Western nations, areca use has enormous impact, both culturally and economically throughout all of South Asia. The commercial value of areca nut production in Taiwan is greater than that of rice [1]. Estimates of chronic areca users range from 200 to 600 million people throughout the world [57]. In the United Kingdom, Asian immigrants often begin areca use in the form of Paan Masala (without tobacco) at ages as low as 10, sometimes switching to preparations with tobacco included once they have established the habit as young adults [58]. In Taiwan approximately 10% of the population above the age of 10 chew betel quid. In urban areas, it is common to see betel quid being sold out of brightly lit glass booths by scantily clad "Betel girls" who deliver it street-side to mainly male customers who drive up to the booths.

Although once a preferred habit of Asian nobility, areca use is now for the most part inversely related to education and income. In spite of the clear evidence for the health risks associated with areca use, in most cultures where use is high, it is tolerated and individuals are not highly motivated to quit [58]. This mirrors the circumstances and attitudes regarding smoking fifty years ago in the U. S. and other Western nations prior to public health campaigns such as those spearheaded by the Surgeon General to alert the public to the health hazards and addictive properties of smoking. In 2004, the International Agency for Research on Cancer, supported by the World Health Organization issued a monograph, "Betel-Quid and Areca-Nut Chewing" [59], which summarized the epidemiological data associating areca use with oral diseases and cancer. Despite appreciation of this health risk [8,60], the klaxons of public awareness have not roused the nations of South Asia to initiate campaigns against areca use and addiction. We have identified a common, and perhaps even synergistic, link between arecoline and the molecular mediators of nicotine addiction that may help put areca addiction on the path to declining social impact that tobacco use has been following for the last fifty years.
